# Supplementary material for: Impact of Nutrition on Short-Term Exercise-Induced Sirtuin Regulation: Vegans Differ from Omnivores and Lacto-Ovo Vegetarians
Source: Nutrients. 2020 Apr 5;12(4):1004. doi: 10.3390/nu12041004 (PMC7230533; doi:10.3390/nu12041004)
Supplement: Supplementary file 1 [file nutrients-12-01004-s001.pdf]

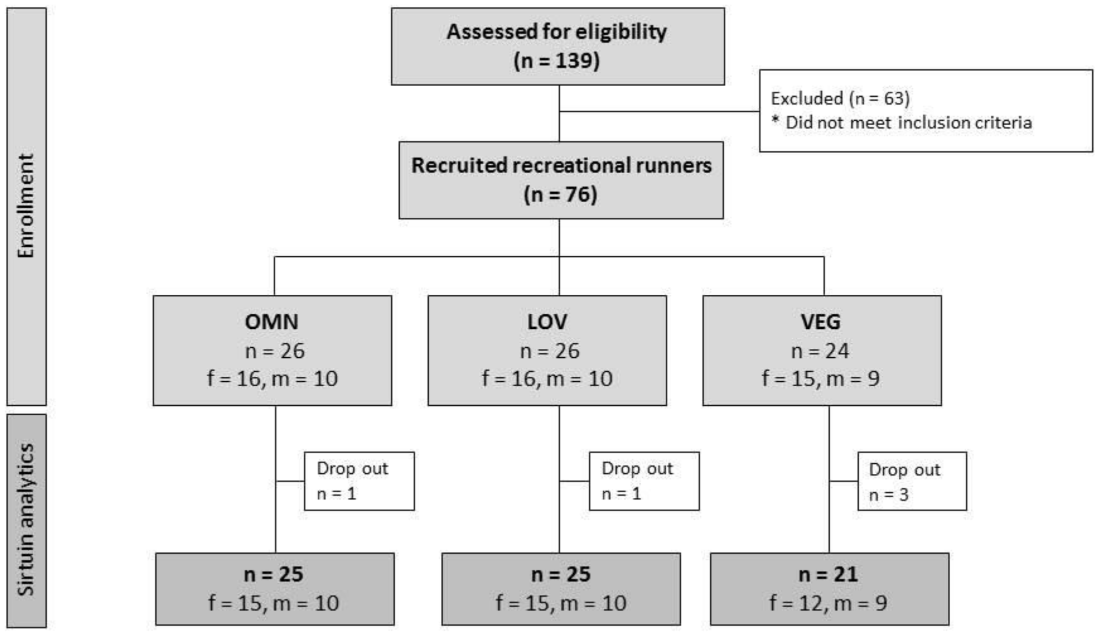

Figure S 1. Flow chart of the study population.

Table S 1. Sequences of qRT-PCR-Primers.

human SUPT20H (forward)	AAC TTT TGC TTG AGA GCC AGC
human SUPT20H (reverse)	TTG CTG CCG ATT CAG AGA GG
human SIRT1 (forward)	CAA CTT GTA CGA CGA AGA C
human SIRT1 (reverse)	TCA TCA CCG AAC AGA AGG
human SIRT3 (forward)	CAG TCT GCC AAA GAC CCT TC
human SIRT3 (reverse)	AAA TCA ACC ACA TGC AGC AA
human SIRT4 (forward)	GCT GTG AGA GAA TGA AGA TGA GC
human SIRT4 (reverse)	CTT GGA AAG GGT GAT GAA GCG
human SIRT5 (forward)	AGT GGT GTT CCG ACC TTC AG
human SIRT5 (reverse)	CAT CGA TGT TCT GGG TGA TG

Table S 2. Correlations of SIRT-activities with different parameters.

Sirtuin	Correlation with	p-value
**SIRT1**	Coffee	0.238
	Flavonoids	0.352
	Polyphenols	0.324
	Vitamin B_{12} in serum	0.411
	Active vitamin B_{12}	0.433
	Glucose	0.416
	Insulin	0.305
	Calories	0.603
	W/kg body weight	0.979
**SIRT3**	Coffee	0.190
	Flavonoids	0.196
	Polyphenols	0.146
	Vitamine B_{12}	0.349
	Active vitamin B_{12}	0.140
	Glucose	0.377
	Insulin	0.090
	Calories	0.897
	W/kg body weight	0.627
**SIRT5**	Coffee	0.388
	Flavonoids	0.067
	Polyphenols	0.382
	Vitamine B_{12}	0.214
	Active vitamin B_{12}	0.075
	Glucose	0.274
	Insulin	0.111
	Calories	0.704
	W/kg body weight	0.623

Statistical analyses were conducted with the Spearman correlation test.
